# “I am not just a place for implementation. I should be a partner”: a qualitative study of patient-centered care from the perspective of diabetic patients in Saudi Arabia

**DOI:** 10.1186/s12913-023-10391-0

**Published:** 2023-12-14

**Authors:** Reeham Ahmed Alkhaibari, Jennifer Smith-Merry, Rowena Forsyth

**Affiliations:** 1 Sydney School of Health Sciences, Faculty of Medicine and Health, The University of Sydney, NSW, Australia; 2 College of Nursing, Taif University, Taif, Saudi Arabia; 3 Centre for Disability Research and Policy, Sydney School of Health Sciences, Faculty of Medicine and Health, The University of Sydney, Sydney, NSW Australia; 4 Cyberpsychology Research Group, Biomedical Informatics and Digital Health Theme, School of Medical Sciences, Faculty of Medicine and Health, The University of Sydney, Sydney, NSW Australia

**Keywords:** Patient involvement, Patient centered care, Saudi Arabia

## Abstract

**Introduction:**

Patient involvement in care is a major component of high quality of care and is becoming recognized worldwide with many beneficial for improving patient outcomes. However, a little is known about patient involvement in the Middle East region and Saudi Arabia in particular.

**Objectives:**

To evaluate patients’ perceptions of their involvement during their interactions with healthcare providers in Saudi Arabia.

**Methods:**

A qualitative exploratory study using semi structured interview was conducted from February 2022 to March 2022. Responses were transcribed and analyzed using a thematic analysis approach.

**Results:**

We conducted seven interviews with patients with diabetes ranging in age from 19 to 69 years old. We identified the following themes:1) patients’ perceptions of their involvement in care, 2) barriers to patient involvement, 3) effective communication, 4) empathy, and 5) culture. We found that patients had minimal knowledge of patient involvement in care.

**Conclusion:**

There is a clear need to improve education and awareness of patient involvement in Saudi Arabia. By educating patients about the possibilities of patient involvement and explaining their role it will make it easier for patients to understand appropriate levels of involvement. In addition, there is a need to understand the patient-centred care culture in Saudi Arabia through establishing frameworks with the focus on culture and patient-centred healthcare delivery.

## Introduction

Patient involvement in care is considered a central pillar of patient centered care (PCC) [1]. The United States-based Institute of Medicine (IOM) highlights PCC as one of the six core elements of achieving quality care along with safety, timeliness, effectiveness, efficiency, and equity [2]. Multiple studies have shown that patient involvement is associated with positive outcomes including improved patient knowledge and health status [3–8]. In order to achieve PCC, Corbett and Ennis [9] emphasized that health providers are required to shift away from the traditional paternalistic paradigm of care where practitioners know best and patients follow their advice unquestioningly. This paradigm shift enables patients to become more knowledgeable, involved, emancipated and therefore able to control and participate in their own healthcare. This shift requires establishing a relationship between patients and health providers that is based on negotiation and compromise until they reach an agreement to implement the best option of care [10]. Couët et al. [11] argue that patient involvement does not only rely on the healthcare provider and therefore it is unrealistic to consider that health providers alone hold the responsibility to involve patients; patients and communication practices also play a significant role. Engagement and interaction with providers represents the primary opportunity for patients to impact medical decisions and the course of treatment [12]. Patients can also enhance their comprehension of the medical process, particularly the rationale behind treatment and follow-up procedures [12]. PCC therefore relies on effort and interaction between patients and providers in the context of appropriate communication, where all need to work together to successfully implement PCC.

### Involving patients in care

Internationally, there have been efforts to promote the philosophy of patient involvement in care. For example, the Picker Institute is a not-for-profit organization, located in the United States, that collaborates with patients, family, health providers and policymakers to improve patient experience in healthcare. It promotes an approach to patient experience based on the eight dimensions of PCC: prompt access to care; efficient treatment; quality care and adaptive transparency; patient and family involvement; comprehensible healthcare knowledge and support, mutual decision-making and respect for patient preferences; emotional support, empathy and respect, and awareness of physical and environmental needs [13]. These dimensions provide a guideline for healthcare organizations to implement and, when necessary, improve the delivery of PCC [14]. Another example is the Planetree model, utilized by health organizations worldwide [15]. Through patient and health worker feedback the Planetree model identifies PCC principles including providing compassionate care, establishing therapeutic partnership between patients, family members, and health providers through patient education and recognizing the significance of providing mental and spiritual support to patients [16, 17].

Research shows that there are a number of factors that influence patients’ preferences regarding their involvement in care. A systematic review found that patients want to be engaged in their care [18] but that their preferences might differ based on their demographic and personal characteristics which include gender, health status, level of education and age [19–21]. For example, patients who are female, with better health, have higher levels of education and are younger are more likely to be actively involved in care, in contrast to patients with low socio-economic status, who are severely ill, have low levels of education and are male [20, 22, 23].

### Patient centred care in Saudi Arabia

The plethora of literature focusing on PCC is predominantly from western cultures with minimal research or literature focusing on Middle Eastern countries which means that there is limited understanding of whether and how PCC is practiced in the Middle East and what barriers and facilitators to that practice might be [24]. Existing literature focused on healthcare in the Middle East indicates that patients support their involvement in care [1, 25, 26] and there is a growing interest in PCC in the Middle East. However, there are still many obstacles to delivering PCC and integration of PCC in health systems in the Middle East region is lacking [24].

In Saudi Arabia, patient-centred practice of in some health organizations remains in its infancy due to existing health system priorities being centred on staffing and resources [27]. The global shift to PCC has led the Saudi government to propose transformational goals as part of its *Vision 2030* to improve healthcare, quality of life, healthcare organization and staff accountability to deliver care that is safe, effective, patient centred, timely, and equitable [27, 28].However, a study conducted in Saudi Arabia with hospitalized patients found that they were not aware of their rights to be fully informed of their medical condition and treatment plan [29].

Few studies have specifically explored PCC in Saudi Arabia from the patients’ perspective. [1]. Existing studies that do focus on some aspects of patient perspectives have demonstrated that patients exhibit a preference for involvement in decision-making regarding their medical care [30, 31]. However, some patients still lean towards a paternalistic approach, where medical decisions are predominantly made by healthcare professionals [30]. Interventions to promote PCC culture to improve adherence to treatment plans and therefore better health outcomes are recommended [31]. There is also a need to explore in depth patients’ preferences to be involved in care to implement PCC more effectively in Saudi Arabia [30, 31]. This study addresses the deficit by employing qualitative interviews to explore patient experiences, to provide data for enhancing patient-centred care practices and implementing necessary improvements in Saudi Arabia.

To understand PCC practice we have chosen to focus on a patient group where people need to have ongoing interactions with healthcare providers as part of the management of their health needs. We focus on people living with diabetes as it is an illness that requires ongoing management and medical care, adoption of a healthy lifestyle, and regular health examinations, with interventions more successful with patient involvement in this care [32]. In Saudi Arabia, there are several challenges that affect the quality of diabetes care. These challenges can be characterized as patient factors (which include adherence, compliance, attitudes, beliefs, knowledge, financial resources, and co-morbidity) and healthcare providers factors (associated with their beliefs, attitudes, knowledge, interaction between health professionals and patients, and communication) [33]. People with diabetes may also face a serious range of complications which includes blindness, cardiovascular disease, and neuropathy which might lead to amputation [34] thus, they are often faced with treatment decisions that require urgency [35]. This means that they are already highly likely to have participated in decision making over the course of their condition, but also have to interact with a variety of health providers and can therefore reflect on their level of involvement more broadly [36, 37]. This exploratory study therefore aims to provide detailed experiential data related to the perspectives of people living with diabetes regarding their involvement in healthcare in Saudi Arabia. We examine patient perceptions of PCC to add to address gaps in existing literature related to patient attitudes towards PCC in the Middle Eastern region.

## Methods

### Study design

A qualitative exploratory study was conducted to assess patients’ perceptions of their involvement during interactions with healthcare providers. The study aimed to achieve an in-depth understanding of the social reality of the research participants by exploring their personal experiences of their involvement in care and their experiences of their interactions with healthcare providers [38, 39]. This publication adhered to the Consolidated Criteria for Reporting Qualitative Research (COREQ) [40].

### Setting and participants

The setting of this study was the Diabetic Center at King Abdulaziz Specialist Hospital, one of the referral hospitals offering specialized services in Taif, Saudi Arabia. This hospital was chosen due to its strategic geographical location and patient demographic, as Taif is recognized for having a high prevalence of diabetes [41]. Inclusion criteria for participants were that they were at least 18 years of age, had been living with type 1 or type 2 diabetes mellitus, were currently receiving care at a diabetic center in Saudi Arabia, and could speak and understand Arabic, which is the main language used in Saudi Arabia. Purposive sampling was used as we sought to recruit participants from a range of ages and who had varying lengths of time living with diabetes. As this was an exploratory study, the aim was not for generalizability of findings but to include a study population who could provide in-depth content-rich data based on their experiences. As described by Malterud et al. [42] such a sample provides ‘high informational power’ and therefore lower sample sizes are acceptable for this type of analysis. Participants were initially recruited via a more general online questionnaire (reported elsewhere) focusing on patient provider interactions and perspectives related to PCC. This survey invitation was distributed using WhatsApp which is a common way of communicating with patients in this setting. At the end of this survey participants were asked if they wanted to participate in an interview. An email with the information statement and the consent form was sent to all participants who indicated their interest. Seven participants provided their consent and participated in an interview.

### Ethical approval

The study protocol was approved by the Human Research Ethics Committee of the University of Sydney [2021/530] and from the Saudi Arabia Directorate of Health Affairs Taif Institutional Review Board [HAP-02-T-067 number 596]. Written consent was obtained from participants and all participants were informed that their participation was voluntary. All potentially identifying information was removed from the interview transcripts at the point of transcription and checked by members of the research team to assure that any quotations used in the paper did not inadvertently identify participants. Transcripts were checked by participants who could remove any data that they did not want included in the study.

### Data collection

Semi structured telephone interviews were conducted between February 2022 and March 2022. The interview schedule contained open-ended questions related to patient experiences of their interactions with healthcare providers. An example of the questions asked in the interviews were “How do you define your role in the healthcare?” And “Do you prefer to be involved in making decisions regarding your healthcare? Why/why not?”. Probes were used to elicit more detailed responses when needed. Notes were taken during the interviews. The duration of the interviews ranged from 20 to 51 min, with an average of approximately 34 min. All interviews were conducted in Arabic by the lead researcher (RAA) who is fluent in Arabic and English. After the transcription was completed, all interviews were translated from Arabic into English by the lead author. The anonymized transcripts were reviewed by another native Arabic and English speaker to ensure the translation was accurate. The analysis was conducted on the English-language version of the transcripts, so the other authors could confirm the validity of the data and analysis process.

### Researchers

RAA is a Saudi Arabian female PhD candidate and a lecturer at Taif University, with experience of the Saudi healthcare system, has received training in conducting qualitative research and did not know any of the seven interviewees in advance of the study. JSM is a female Professor with extensive experience in qualitative research and has conducted research previously in Saudi Arabia and other countries in the Middle East. RF is a female senior lecturer with extensive experience of in qualitative research.

### Data analysis

As this was an exploratory study, where we were interested in the phenomenon of patient centred care from the perspective of patients, we conducted an inductive analysis of the data which aimed to draw out the key aspects of their experiences of care. In qualitative studies the validity of research is verified through the criteria of credibility, confirmability, transferability, and dependability [43]. We followed a credible, structured process of data analysis using the thematic analysis process proposed by Braun and Clarke [44] to identify, analyze, and report the patterns from the data. The transcripts were read several times for familiarization before initial codes were developed. After that, similar codes were grouped into potential themes. The next stage, review of the themes, was conducted to ensure that themes formed a coherent pattern. Once the themes were adequately identified we created a thematic map. Lastly, ongoing analysis was conducted to define the essence of each theme.

The lead author discussed and reviewed all steps of the analysis with other authors. To ensure the credibility of the findings we employed member checking with transcripts shared with participants for them to review and to add anything they wanted to clarify further. To enhance transferability, we provided background information about each of the participants. In addition, we documented an audit trail of all the processes of data collection and data analysis throughout the study to ensure confirmability. Dependability was ensured through using an interview schedule, audio recording and full transcription of the interviews. As this aimed to be an exploratory study utilizing Braun and Clark’s [44] method of analysis saturation was not an aim of the data collection [45]. Instead we aimed to provide an insight into understandings of patient-centred care from the participants via in-depth interviews [38, 42]. The findings section below is ordered in relation to the main themes identified during the inductive coding process described above.

## Findings

### Participant demographics

Table 1 outlines the participant demographic characteristics. The study participants consisted of three females and four males, with three aged 18–39 and four aged over 40. Five participants reported they had been diagnosed with diabetes for more than 10 years with the other two ranging from 2 to 5 and 6–10 years since diagnosis. The majority of participants indicated that their highest level of qualification was a bachelor’s degree.

**Table 1:** Participants’ demographic characteristics

Participants ID	Gender	Age	Highest qualification	Duration living with diabetes
P1	Male	40–54	Undergraduate degree and above	10 years and more
P2	Male	40–54	Undergraduate degree and above	10 years and more
P3	Female	18–39	Undergraduate degree and above	2–5 years
P4	Female	18–39	Undergraduate degree and above	10 years and more
P5	Male	55–64	Undergraduate degree and above	10 years and more
P6	Male	65–74	Undergraduate degree and above	10 years and more
P7	Female	18–39	Undergraduate degree and above	6–10 years

### Patients’ responses

The study findings explore the experiences of the participants and how they viewed their involvement in decision making. The interviews were mutual interactions where both the lead researcher and the research participant were engaged in dialogue. A description of the participants’ situations is provided below in order to provide context to their individual responses:

P1: Through the interview it was clear that P1 was irritated about how he had been treated by health providers and his voice was full of hope that his participation in this research would make a positive impact on patient involvement in care.

P2: This participant passionately demanded better care and was desperate to have a healthcare provider with full knowledge of his condition who would seek to obtain more information from him.

P3: During the interview P3 indicated her disappointment with her recent interaction with a healthcare provider who does not speak Arabic. However, she appeared to be more reluctant to express negative opinions about healthcare providers.

P4: This participant was upset and disappointed with her healthcare provider for ignoring her and preferring to interact with her parents rather than interacting with her. During the course of the interview her tone shifted as she started describing her favorite healthcare provider, who is a female, as the healthcare provider was the only one who would actively listen to her and allow her to express her thoughts.

P5: This participant’s voice was calm and full of gratefulness. He positively recalled his healthcare provider’s positive attitude when she connected him with other healthcare providers to keep following up on his other health issues. He was touched by this personalized approach. He hoped that a new generation of healthcare providers would participate positively to improve patient-provider relationships.

P6: In his interview P6 sounded conflicted or confused as he believes that there is no relationship between healthcare providers and patients, however later he explained the relationship should be based on what a patient needs and what the doctor has to offer. He was hesitant to express negative opinions especially when he was asked about the impact of gender roles.

P7: During the interview P7 sounded defeated because for a long time she had never experienced any involvement from her previous healthcare providers and therefore was hesitant to get involved in her care or share concerns with her provider. However, more recently her experience changed as she was assigned to a new healthcare provider who motivated her and encouraged her to voice her opinions. She prefers to have a friendship with healthcare providers as she believes it is the way to facilitate her interactions with them.

### Qualitative themes

Participants shared their experiences of involvement in care and what they believe is their role in the healthcare system. We identified five key themes: patients’ perceptions of their involvement in care, barriers to patient involvement, effective communication, empathy, and culture. Elements of these themes can be seen in Table 2 with each expanded on in turn within the text below.

**Table 2:** Themes and categories from thematic analysis

Themes	Categories
Patients’ perceptions of their involvement in care	Patient involvement
	Patient role in care
Barriers to patient involvement	Patient related barriers
	Health provider related barriers
	Environmental related barriers
Empathy	Patient empathy toward healthcare providers
Effective communication	Communication skills
	Health provider characteristics
Culture	Gender role
	Cultural norms

### Patient perception of their involvement in care

Patients displayed favorable views towards patient involvement with regard to their own health. For example, one participant stated: “I support it [patient involvement] in order to be a partner in the outcome” (P6). Participants varied however as to the extent to which they wanted to be involved in care. Participants commented on the importance of their own expertise of diabetes as the basis of their role for participating in decision making.

“I am the one who will practice the treatment. Therefore, the opinion of the patient should be taken in everything …. In my opinion I should be a partner in the treatment. I am not just a place for implementation. I should be a partner. I am the most knowledgeable person in my case, and I should have full information about the disease and the treatment plan and how it works. What is my role in achieving progress in it? … the patient should be a participant deciding on a treatment plan” (P1).

“Because I am the patient … the doctor studied the disease, but he does not feel it. I mean, he did not live with it personally. I am the one who can describe my experience, and make a decision, this is for my benefit…I must interact with the doctor so that the decision going to be useful for me, not just that I have to comply with it.” (P4).

Other participants stated that they preferred to have a minimal role in their care and mostly rely on the healthcare providers’ medical knowledge for decision making. Example comment was:

“ Some decisions only the doctor can make…I follow his decision because he has experiences with people before me…I may express my opinion, but I respect the doctor’s opinion“ (P2).

One participant was clear that she did not want to be involved in care and instead preferred to rely on doctors’ knowledge however, she wanted to be involved to improve her own lifestyle. She commented:

“My participation, I mean, I will not participate in general, I mean, almost the doctor knows everything, but some daily practices, some sleep regime, for example, I mean, I feel this thing belongs to me” (P3).

Whilst some participants claimed that they supported the idea of patient involvement in care, when questioned about their roles or involvement in healthcare the interviewees demonstrated minimal knowledge of what would generally be associated with the key attributes of patient involvement in PCC, for example within the Planetree or Picker Institute models discussed earlier. For example, one participant stated:

“The patient’s role is helping the doctor by complying with the treatments and following diets, as he [healthcare provider] says by exercising with the treatment… [the] patient must take care of himself. The most important thing is to follow the instructions that the doctor says” (P2).

Another participant commented:

“I follow the things the health provider instructs for example, appointments, using treatment, adhering to the health provider’s recommendations… the most important thing is to adhere to the things that the doctor has recommended.” (P5).

When asked about the role of healthcare providers, participants likewise stated that it was to “listen to the patient and asking questions” (P5) or to “diagnose patients and explain the treatment to the patient” (P6). The role of patients in these responses was more associated with complying with healthcare provider directions and treatment, which indicates a passive patient rather than an active one. Another participant addressed the absence of patient involvement practices in care in the healthcare system in Saudi Arabia, stating that the “…patient role is marginalized. The patient mostly doesn’t have a role” (P1).

In summary, it appeared that most of the diabetic patients wanted to be involved in their care. However, for some, the way in which they articulated their role in care was to comply with healthcare provider instructions and be involved in adopting a healthier lifestyle. Hence, from these responses it appears that some participants were unaware of their role in care and therefore to the concept of patient involvement.

### Barriers to patient involvement

Despite the support for patient involvement in care expressed by participants, they expressed several barriers when interacting with healthcare providers. These barriers were (i) patient related; (ii) healthcare provider related; or (iii) environment related.

#### Patient related barriers

Participants expressed that they were concerned about interacting with the healthcare providers. One participant commented:

“*…*I am a person; … I do not like to be ignored by anyone… my treatment plan was built by myself based on the doctor’s words and the information I have, so I do not rely on his [healthcare provider] treatment plan. I just try to follow the tests and try to follow up on the sugar levels with them, so I consider it part of my treatment plan” (P1).

One female participant also commented that she did not like to engage with the doctor during consultations but preferred to communicate with the healthcare provider through the diabetes educator.

“I am not the type to express …. I mean the doctor tells me what he has… that if he asked me, I would answer, but if he for example, asked me why I use this medication, or I tell him that I don’t want to use this medication … I may tell the health educator and she delivers it to the doctor but he doesn’t interact with me” (P4).

These quotations suggest that patient behavior can limit patient involvement in care due to hesitation to ask questions, or patients prefer to interact with the health educator rather than the doctors.

#### Health provider related barriers

Most participants highlighted that the professional conduct of the healthcare provider was a contributing factor which could obstruct patient involvement in care. One participant commented that the lack of professional ethics and humanity in healthcare when healthcare providers interact with patients acts as a barrier to PCC. He stated that “Dealing with compassion and mercy I see it as non-existent” and “…professional ethics almost do not exist…” (P1). Other participants described the types of healthcare provider behaviors which act as barriers for their involvement in care as: dismissing patient concerns, a lack of eye contact, language barriers, and lack of empathy.

Participants expressed the view that healthcare providers fail to encourage patients to share or ask questions. For example:

“I expected him [health provider] to ask me … I had so many questions and I felt that he will ask me about them [questions]… he read the test [results] and wrote the treatment and that’s it.” (P3).

Another participant believed that while healthcare providers ask questions, they do so to establish the treatment plan rather than making the patient involved in care.:

“Sometimes there are questions…but the questions are not for participation … He [healthcare provider] only takes the information to decide” (P1).

This might indicate that the current practices of communication and patient involvement in care are more physician centered.

One participant provided an example of poor patient engagement, where their healthcare provider seldomly paid attention or looked at him during the consultation because the healthcare provider focused on their computer, stating:

“There was no meeting [eye contact] between me and her [health provider] … [discussing] that the tests were good…. her attention was on the computer” (P2).

Several patients highlighted a lack of empathy by healthcare provider toward the patient as a barrier to patient involvement in care. For example,

“The humanitarian and religious side of dealing with compassion and mercy do not exist. These are financial works that they carry out to earn salaries….” (P1).

Another described their view of empathy in healthcare interactions in the following way:

“…I want him [health provider] to know the path of my disease and interact with me as if he is talking to a friend … so that he knows everything about me … to let me talk.” (P2).

This shows that health providers’ lack of empathy would negatively influence patient involvement in care as they dismiss patients’ need to interact and share their concerns.

Another factor contributing to the extent of patient involvement in healthcare is a lack of up-to-date knowledge by the provider, with one participant reporting:

“Sometimes the patient’s information, not medical information, but information about the disease that is equal to the doctor, they [health providers] don’t accept it, even in research we [patients] have knowledge about … some doctors don’t keep themselves up to date, so they are outdated” (P1).

Here the patient’s differing perspectives on current knowledge with respect to diabetes was not valued and discussed, which undermines the relationship. The same participant believed that he was treated by the healthcare provider in a way where patients were not viewed as individuals but rather as part of a job to gain financial benefits (P1). Another participant commented:

“There are doctors, I mean, … his attitude, [is] that I am [the healthcare provider] here to do a job to do what I [healthcare provider] have to do and that’s it.” (P7).

These experiences show that behaviors by healthcare providers impact patient involvement in care and that healthcare providers have the opportunity to improve patient involvement by having empathy towards patients and actively encouraging them to share their concerns. Additionally, based on the participants’ perspectives, most of the barriers reported were associated with the health provider interpersonal skills, which might affect patient perceptions of their involvement in care.

#### Environment related barriers

Participants commented on a lack of continuity of care and other elements of healthcare operations that impacted on patient-centered care. For example, seeing different healthcare providers at every appointment or high clinician workloads that limited the time available to them to interact with the healthcare provider. For example, participants stated:

“If they [healthcare providers] were not busy, they would sit with you [patient] … and start questioning you and see what you are missing and what you need. If they [healthcare providers] were busy [they say] ‘we will communicate with you through WhatsApp’ … and they will not respond to you…” (P3).

“…every appointment is with a doctor or [a female] doctor. I feel that you [patient] want to get it over with. You do not have a meeting with someone who knows you and is close to you.… the communication process, it is quite difficult” (P2).

Many participants also reported long wait times for appointments where it would take four to six months to make an appointment with the healthcare provider.

“[the healthcare provider said] that your next appointment will be after six months. This was the first time I took an appointment after six months I felt it is a little faraway and I said, ‘that it is fine’.” (P3).

During long periods of waiting for appointments one participant expressed the challenge they had in getting in contact with the healthcare provider to have their needs addressed:

“You [patient] cannot communicate with him [healthcare provider] with anything no matter what happens to you [outside of their scheduled appointment] … I mean, there is no means of communication at all. They used to have a groups chat, but it was cancelled.” (P5).

This indicates that other barriers might be embedded within the Saudi healthcare system that contributes to the long waiting time and hence work as barriers to patient involvement in care.

### Empathy

Despite their individual complaints, the majority of participants spoke about having empathy towards their healthcare providers. They displayed an understanding of how busy the work environment was and therefore the heavy pressure faced by their health providers. One participant stated:

“[healthcare providers are] bearing the pressure that occurs throughout the day, so when I come, I do not want to say that he [healthcare provider] does not treat me well. Even if, for example, he [healthcare provider] scolded me or ignored me at the same time for me, its fine … he is a person who is experiencing stress and going through a stage that he has many patients… I would understand that they [healthcare providers] have many patients, and I am not the only patient.” (P4).

Another participant stated that there is a:

“…lot of pressure on them [healthcare provider]. He [healthcare provider] cannot give you [patient] enough time in order to understand you and listen to what you have because I mean I feel for them that he [healthcare provider] is stressed and that he has so many patients” (P5).

### Effective communication

Effective communication is viewed as an integral component of patient provider interactions. Participants stated that healthcare providers asked them about their conditions and encouraged them to express their concerns. Participants spoke about experiences of good practitioner communication. For example:

“…. I used to meet doctors, frankly. I mean, I can’t praise them enough. There was an eye contact. They asked me about my condition“ (P3).

“She [healthcare provider] asked about me, ‘what do you [patient] have? what are the things that you complain about?’…. I told her I have the heart condition …. and she helped me to get comfort and treatment and she connected me …. she connected me to another doctor in another clinic” (P5).

Another participant stated that good communication skills were essential to encouraging and motivating patients to share their concerns during a consultation:

“He really left room for me to speak, I mean, I think on the last visit, I could have talked for about half an hour” (P7).

One participant commented that having a “friendship” with the healthcare provider would simplify the communication process. She stated:

“If [the relationship] is not formal then everything is fine…. he [healthcare provider] has no problems to hear from you [patient] all the time and gives solutions. If it was a formal relationship you [patient] will not be able to discuss with him [healthcare provider] because he doesn’t like to talk so much and doesn’t want problems so you will remain silent but if it was almost as we said that it is a friendship or something like that, this for me is more important than the quality itself.” (P7).

Patients’ descriptions of positive healthcare provider characteristics during interactions were focused on actively listening to patients, positivity, patience, and the ability of providers to put themselves in the place of the patient. The following quotations provide an account of the ideal practitioner from the participant’s perspective:

“First, to be smiling, cheerful … to accept the patient as he is, to listen to him … to be familiar with the aspects of the disease that he treats, and to be aware of the capabilities and tools he possesses …in the health facility in which he works, so that he is dealing with reality…. to be realistic with the patient …earns his [patient’s] affection and their interactions would be based on honesty” (P6).

“To put himself in the place of the patient I mean, …. To feel the patient’s feelings and make him feel …. That when [the patient] is talking, he is not wasting time…” (P5).

Participants variously emphasized that healthcare providers should have “patience”, “endurance”, “understanding”, should “support patients”, “put themselves in [the] patient’s place”, “ask more questions”, “encourage patients to talk”, and use “motivational phrases” which would influence the patient to actively participate in their care.

### Culture

Several participants expressed factors related to Saudi Arabian culture related to gender and cultural that influenced their interactions with their healthcare providers.

#### Gender role

The role of gender in impacting patient provider interactions differed among the participants. A few participants commented on their preference to receive care from a healthcare provider of the same gender. This view could reflect cultural practices. One male respondent expressed the need to interact with a male healthcare provider when he wanted to discuss relationship issues:

“There is a big difference……. The culture of society affects. Yes, I cannot say some things, for example, except when I am with a [male] doctor … I mean, it is possible in matters related to my relationship with the other party [wife]” (P2).

Another female participant stated that in general she was more comfortable to have a female healthcare provider under the assumption that she can bond with her better due to their shared gender. She commented:

“Yes, it makes a difference when the healthcare provider is a female, I can interact with her and deal with her that she is the same gender. She can understand me more when I explain, I will be comfortable that I explain and talk to her …. While a man, I will not be able to interact with him” (P4).

However, this view was not shared by all respondents as one female participant commented:

“For me, I do not feel any difference, in the end, they [healthcare providers] are all performing the same mission and message” (P3).

A similar view was expressed by another male participant who indicated that the gender of the healthcare provider is not important as long as the healthcare provider actively involves patients in a conversation. Another also commented that what matters is the healthcare provider’s attitude, which affects patient provider communication. He stated:

“I do not [see] any difference. The same ethics, the same method, and the same treatment, …. I mean … they teach each other a way of interacting [it is] the same thing, whether the healthcare provider is male or female.” (P1).

For these participants the gender of the healthcare provider was not a significant factor, with provider behavior instead the most significant factor in facilitating patient involvement.

#### Cultural norms

In Saudi culture, which is strongly influenced by Islam, the young are strongly expected to respect their elders [46, 47]. This is reflected in the views of one female participant, who commented:

“… I always respect that he [healthcare provider] is older than me and is talking to my mother. I mean, I just listen to their interaction …” (P4).

Saudi Arabia culture is also collectivist rather than individualist, however, only one participant reported a form of family involvement in care. For this participant her needs were dismissed by her healthcare providers who instead involved her family in decision making about her care. She stated,

“They [healthcare providers] did not give me the freedom to speak… the whole questions weren’t directed at me … the questions were directed to my mother and father and not me. I tried to explain to them that I understand the diabetes and I have been living with diabetes for 19 years therefore I understand my condition, but they prefer to listen to my mother instead of me” (P4).

This suggests that the Saudi Arabian culture have a significant influence on patient-provider interaction and hence patient involvement in decision making.

## Discussion

As far as we are aware this is the first paper to explore patient perceptions of their involvement in healthcare in Saudi Arabia. The main themes emerging from this exploratory study reveal important insights about the nature of patient involvement in Saudi Arabia by providing qualitative data from in-depth interviews with patients.

### Mis-conceptualization of patient involvement and patient role

Our study showed that most participants had minimal knowledge of patient involvement in care. This was consistent with previous studies in other contexts that have investigated patient perceptions of involvement in decision making in cancer [48] and diabetes [49]. Those studies found that patients had limited knowledge of concepts of shared decision making and that their preferences were more focused on sharing information rather than sharing decision making [48, 49]. There are several factors that contribute to patients’ minimal knowledge of their role in care. First, PCC is considered new in some healthcare organizations in Saudi Arabia [27], with patients lacking understanding of their rights to be involved in care [29]. It may also be due to the nature of power dynamics between patients and healthcare providers in Saudi Arabia where patients perceive healthcare providers as authoritative figures whose words should be trusted in an unquestioning way [50].

In our study patient willingness to participate in healthcare decision making was affected by their type of involvement, however in general participants were found to take a minor role in decision making and rely on health providers’ expertise. This finding reflects patient involvement norms beyond the Middle East and North Africa (MENA) region. For example, a national survey of 2765 people in the United States of America aiming to evaluate preferences for participating in decision-making found that 96% preferred to have a choice and to be asked for their opinion [51]. However, participants’ responses varied in the degree to which they would rely on the healthcare provider’s medical knowledge. About 44% of participants preferred to depend on the healthcare providers’ medical knowledge rather than exploring information themselves, and 52% preferred to leave final decision-making to their health provider [51]. In our study we found that participants preferred to have an active role in behavioural decisions such as adopting a healthy lifestyle (e.g., decisions about diet and exercise). These results are in line with the study by Mansell, et al. [52], which investigated whether the type of illness and the nature of decision making predicts patient preferences to be involved in decision making. The study found that patients preferred to be actively involved in major decisions such as surgery and in behavioural change a decisions such as diet and exercise while they preferred to be less involved in minor decisions such as ordering blood tests.

Previous literature has emphasized the importance of having mutual interactions between patients and healthcare providers where information is shared between them as this gives patients the opportunity to feel in control and responsible for their care, and therefore involved in their care [53–56]. There is an inherent power imbalance to the relationship between healthcare providers and patients which derives from the vulnerability of patients who come seeking help, in relation to the healthcare provider’s expert knowledge [57]. There is also a contributing role for culture, where patient involvement may be further limited by the privileged role health practitioners hold in some societies in the MENA region, such as Pakistan, Jordan, and Saudi Arabia [50, 58–60]. In the current study participants showed support for the concept of patient involvement but described their role as predominantly involving information provision to healthcare providers and compliance with healthcare provider instructions. This misunderstanding of the full dimensions of patient participation, as described by the models outlined earlier, points to the importance of increasing awareness of what and how patients in Saudi Arabia can be active participants in care. Increasing knowledge of patient participation and the benefits associated with it, which include improving the level of self-care behaviors, adherence to medications, and overcoming illness related stress and anxiety [5, 61], could in turn positively influence expectations and preferences to be involved [62, 63]. In addition, health organizations in Saudi Arabia need to enable this within a supportive environment in which health providers have a positive attitude and encourage patients to take an active role in care which would, in turn, increase patient involvement in care [48].

### Barriers to patient involvement

Our findings show that healthcare provider communication style can be an important enabler or barrier to person centred care. Effective communication has been clearly established as an essential element that health providers need to practise in order to provide high-quality person-centred care [64, 65]. Examples of effective communication skills are asking open ended questions, active listening, attentiveness to patients’ needs and giving patients time to respond [64, 66, 67]. Participants in this study felt that healthcare providers needed to ask questions and motivate patients to express their needs and concerns. However, according to our participants, health providers often failed to practise these features of effective communication. This finding is supported by research from the MENA region and elsewhere which has shown that most of the reported barriers to patient centred care are focused on healthcare provider behaviour [68–70]. Problematic behaviours identified in other literature includes limited eye contact and dismissing patient concerns [68–70].

Our study found that participants preferred a healthcare provider who shows empathy towards them and listens to their needs. This is consistent with previous studies which have reported that patients prefer healthcare providers who have a positive attitude expressed through actions such as smiling and expressing humour during interactions [71]. However, participants in our study felt that there was a lack of empathy towards them by healthcare providers, including healthcare providers viewing them as just a part of their work rather than as a human being. To address this providing medical students and healthcare providers with empathetic communication skills has been identified as important for positive patient outcomes [72–74]. Our findings suggest that more emphasis should be placed on the development of supportive and therapeutic relationships between patients and healthcare providers in Saudi Arabia in order to establish a role for patients in their own healthcare. Moudatsou et al. [75] reported that healthcare providers find it challenging to be empathetic due to factors which include high patient load and lack of time provided to spend with the patient. Our results reveal that patients have empathy towards their healthcare providers, showing that patients understand the work burden associated with healthcare work environments. This may be as a result of cultural perceptions of healthcare providers in the Saudi community where healthcare providers hold an authoritative position and should be respected and trusted [50].

In Saudi Arabia, culture is strongly influenced by Islam as the Islamic religion dictates the way of life [47]. Gender is culturally constructed in this context and therefore some participants in this study preferred healthcare providers of the same gender in order to receive healthcare that was centred on their needs. This reflects findings of previous research that highlights the significance of providing services in accordance with gender-based norms in relation to patient-provider relationships [76, 77]. In this study one participant also spoke about healthcare providers preferring to involve their family in decision making rather than involving the patient themself. This attitude might be as a result of healthcare provider belief that families have a full knowledge of patient health status [78, 79]. This finding supports previous research which has reflected on the role of family in patient care in the MENA region [58, 60, 70]. According to Hofstede, et al. [80], the nature of culture and power distribution in a society influences the practice of PCC. Hence, it appears that the practice of PCC in Saudi Arabia is influenced by an unequal distribution of power and dependent collectivism. Consequently, there is a need to understand and build PCC culture in Saudi Arabia through establishing frameworks with a central focus on culture [81] .

The findings of this study show that there is still much to do to educate patients about their involvement in care in Saudi Arabia. Consequently, health organizations in Saudi Arabia need to work more to educate both patients and healthcare providers in order to achieve optimal PCC. The Ministry of Health should explore the development of a culturally sensitive patient-centred care model that aligns with the values and norms of Saudi Arabian culture. This research underscores the importance of enhancing patient education regarding their active role in their own care, while also emphasizing the significance of equipping healthcare providers with proficient interpersonal skills to ensure effective implementation. This approach could significantly contribute to the enhancement of healthcare quality within the country. Further research building on this exploratory research is needed to determine how patients view their role and their involvement in care, to examine what factors influence their involvement in Saudi Arabia and to develop relevant models based on this, which can then be put into practice in improving the practice of PCC in Saudi Arabia.

### Study limitations

Our data is an exploratory study which has provided deep understanding of patient perspectives on patient involvement in healthcare in Saudi Arabia. In doing so it provides deep and personal reflections but is limited by only representing the views of diabetic patients who received care in the diabatic centre at King Abdul Aziz Specialist Hospital. Given this limitation we recommend a national investigation regarding public awareness of the role of patients in care in Saudi Arabia. As qualitative research is focused on opinions and judgments based on personal experiences rather than categorical results, future work should expand on this exploratory study by bringing in a larger number of participants from different disease groups, and their carers, and contrasting patient perspectives with those of their healthcare providers regarding the challenges of practising patient centred care.

## Conclusion

This study explored current practices of patient involvement in healthcare from the perspective of diabetes patients in Saudi Arabia. Based on the results reported, there is a clear need to increase both patient and healthcare provider education and awareness of patient involvement in Saudi Arabia. By educating patients about the possibilities of patient involvement and explaining their role it will make it easier for patients to understand appropriate levels of involvement. On the other hand, there is a need to provide healthcare providers with explicit training on PCC and interpersonal skills to facilitate patient involvement in care. The findings of the current study have potential implications for improving the quality of care in Saudi Arabia through shaping education and training for both patients and healthcare providers regarding patient involvement and interpersonal skills.

## Data Availability

Due to the ethical requirements of the study and the need to maintain confidentiality for the participants, the full transcripts which constitute this research data are not available for sharing.

## Abbreviations

PCC: Patient centered care
IOM: Institute of Medicine
MENA: Middle East and North Africa

## References

[1] Alhaqwi A, Aldrees T, Alrumayyan A, Alfarhan A, Badri M (2016). Patient’s desire and preference for provision of information toward greater involvement in shared care. Saudi J Med Med Sci.

[2] Richardson WC, Berwick DM, Bisgard J, Bristow L, Buck C, Cassel C (2001). Crossing the quality chasm: a new health system for the 21st century.

[3] Greene J, Hibbard JH (2012). Why does patient activation matter? An examination of the relationships between patient activation and health-related outcomes. J Gen Intern Medicine: JGIM.

[4] Griffin SJ, Kinmonth A-L, Veltman MWM, Gillard S, Grant J, Stewart M (2004). Effect on health-related outcomes of interventions to alter the interaction between patients and practitioners: a systematic review of trials. Ann Fam Med.

[5] Peimani M, Nasli-Esfahani E, Sadeghi R (2020). Patients’ perceptions of patient–provider communication and Diabetes care: a systematic review of quantitative and qualitative studies. Chronic Illn.

[6] Rao JK, Anderson LA, Inui TS, Frankel RM (2007). Communication interventions make a difference in conversations between Physicians and patients: a systematic review of the evidence. Med Care.

[7] Stewart M, Brown JB, Donner A, McWhinney IR, Oates J, Weston WW, Jordan J (2000). The impact of patient-centered care on outcomes. J Fam Pract.

[8] Ulin K, Malm D, Nygårdh A (2015). What is known about the benefits of patient-centered care in patients with Heart Failure. Curr Heart Fail Rep.

[9] Corbett LQ, Ennis WJ. What do patients want? Patient preference in wound care. Mary Ann Liebert, Inc. 140 Huguenot Street, 3rd Floor New Rochelle. NY 10801 USA; 2014.

[10] Sandman L, Munthe C (2010). Shared decision making, Paternalism and Patient Choice. Health Care Anal.

[11] Couët N, Desroches S, Robitaille H, Vaillancourt H, Leblanc A, Turcotte S, Elwyn G, Légaré F (2015). Assessments of the extent to which health-care providers involve patients in decision making: a systematic review of studies using the OPTION instrument. Health Expect.

[12] Greenfield S, Kaplan S, Ware JE (1985). Expanding patient involvement in care: effects on patient outcomes. Ann Intern Med.

[13] Principles of Person Centred Care. [https://www.picker.org/about-us/picker-principles-of-person-centred-care/].

[14] Rathert C, Wyrwich MD, Boren SA (2013). Patient-centered care and outcomes: a systematic review of the literature. Med care Res Rev.

[15] Frampton S, Guastello S, Brady C, Hale M, Horowitz SM, Smith SB, Stone S. Patient-centered care: improvement guide. 2008.

[16] Frampton SB (2001). Planetree patient-centered care and the Healing arts. Complement Health Pract Rev.

[17] Frampton SB, Gilpin L, Charmel PA. Putting patients first: Designing and practicing patient-centered care. 2003.

[18] Chewning B, Bylund CL, Shah B, Arora NK, Gueguen JA, Makoul G (2012). Patient preferences for shared decisions: a systematic review. Patient Educ Couns.

[19] Arora NK, McHorney CA. Patient preferences for medical decision making: who really wants to participate? Medical care 2000:335–341. 10.1097/00005650-200003000-00010 10718358

[20] Flynn KE, Smith MA, Vanness D (2006). A typology of preferences for participation in healthcare decision making. Soc Sci Med.

[21] Ryan J, Sysko J (2007). The contingency of patient preferences for involvement in health decision making. Health Care Manage Rev.

[22] Benbassat J, Pilpel D, Tidhar M (1998). Patients’ preferences for participation in clinical decision making: a review of published surveys. Behav Med.

[23] Murray E, Pollack L, White M, Lo B (2007). Clinical decision-making: patients’ preferences and experiences. Patient Educ Couns.

[24] Alsulamy N, Lee A, Thokala P, Alessa T (2021). Views of stakeholders on factors influencing shared decision-making in the Eastern Mediterranean Region: a systematic review. East Mediterr Health J.

[25] Akkafi M, Sajadi HS, Sajadi ZS, Krupat E (2019). Attitudes toward patient-centered care in the Mental Care Services in Isfahan, Iran. Commun Ment Health J.

[26] Schattner A, Bronstein A, Jellin N (2006). Information and shared decision-making are top patients’ priorities. BMC Health Serv Res.

[27] Al-Sahli B, Eldali A, Aljuaid M, Al-Surimi K (2021). Person-centered care in a Tertiary Hospital through Patient’s eyes: a cross-sectional study. Patient Prefer Adherence.

[28] Ahmad S, Singh J, Kamal MA, Shaikh ZM (2020). Person-centered care design with reference to healthcare outcomes in Saudi Arabia: an overview. Am J Civ Eng Archit.

[29] Almoajel AM (2012). Hospitalized patients’ awareness of their rights in Saudi governmental hospital. Middle-East J Sci Res.

[30] AlHaqwi AI, AlDrees TM, AlRumayyan A, AlFarhan AI, Alotaibi SS, AlKhashan HI, Badri M (2015). Shared clinical decision making: a Saudi Arabian perspective. Saudi Med J.

[31] Aljaffary A, Alhuseini M, Rayes SA, Alrawiai S, Hariri B, Alumran A (2020). The OPTION scale: measuring patients’ perceptions of Shared decision-making in the Kingdom of Saudi Arabia.(ORIGINAL RESEARCH). J Multidisciplinary Healthc.

[32] Nam S, Chesla C, Stotts NA, Kroon L, Janson SL (2011). Barriers to Diabetes management: patient and provider factors. Diabetes Res Clin Pract.

[33] Almutairi KM (2015). Quality of Diabetes management in Saudi Arabia: a review of existing barriers. Arch Iran Med.

[34] World Health Organization. Definition, diagnosis and classification of Diabetes Mellitus and its Complications: report of a WHO consultation. Part 1, diagnosis and classification of Diabetes Mellitus. In.: World health organization; 1999.

[35] McBride E, Hacking B, O’Carroll R, Young M, Jahr J, Borthwick C, Callander A, Berrada Z (2016). Increasing patient involvement in the diabetic foot pathway: a pilot randomized controlled trial. Diabet Med.

[36] Entwistle V, Prior M, Skea ZC, Francis JJ (2008). Involvement in treatment decision-making: its meaning to people with Diabetes and implications for conceptualisation. Soc Sci Med.

[37] Funnell MM (2004). Patient empowerment. Crit Care Nurs Q.

[38] Ritchie J, Lewis J, Nicholls CM, Ormston R (2013). Qualitative research practice: a guide for social science students.

[39] Maxwell JA. Qualitative research design: an interactive approach. Sage publications; 2012.

[40] Tong A, Sainsbury P, Craig J (2007). Consolidated criteria for reporting qualitative research (COREQ): a 32-item checklist for interviews and focus groups. Int J Qual Health Care.

[41] Abdelhafeez S, Al-Swat H, Babiker S, Nasir O, Eisa B. The prevalence of Diabetes Mellitus in Taif Region, Saudi Arabia during 2013–2014. International Journal of Science and Research (IJSR); 2018.

[42] Malterud K, Siersma VD, Guassora AD (2016). Sample size in qualitative interview studies: guided by information power. Qual Health Res.

[43] Lincoln YS, Guba EG (1985). Naturalistic inquiryBeverly Hills.

[44] Braun V, Clarke V (2006). Using thematic analysis in psychology. Qualitative Res Psychol.

[45] Braun V, Clarke V (2021). To saturate or not to saturate? Questioning data saturation as a useful concept for thematic analysis and sample-size rationales. Qualitative Res Sport Exerc Health.

[46] Ahmed AS. Discovering Islam: making sense of Muslim History and Society. Routledge; 1988.

[47] Saleh Al Mutair A, Plummer V, Paul O’Brien A, Clerehan R (2014). Providing culturally congruent care for Saudi patients and their families. Contemp Nurse.

[48] Sainio C, Eriksson E, Lauri S (2001). Patient participation in decision making about care: the cancer patient’s point of view. Cancer Nurs.

[49] Peek ME, Quinn MT, Gorawara-Bhat R, Odoms-Young A, Wilson SC, Chin MH. How is shared decision-making defined among african-americans with Diabetes? Patient education and counseling 2008, 72(3):450–8. 10.1016/j.pec.2008.05.018 PMC3339628 18684581

[50] Banaser M, Stoddart K, Cunningham N. A qualitative study of patient satisfaction in oncology wards setting in Saudi Arabia. Research and Reviews: Journal of Nursing and Health Sciences 2017, 3(3):85–97.

[51] Levinson W, Kao A, Kuby A, Thisted RA (2005). Not all patients want to participate in decision making: a national study of public preferences. J Gen Intern Medicine: JGIM.

[52] Mansell D, Poses RM, Kazis L, Duefield CA. Clinical Factors That Influence Patients’ Desire for Participation in Decisions About Illness. Archives of internal medicine (1960) 2000, 160(19):2991–2996. 10.1001/archinte.160.19.2991 11041908

[53] Eldh AC, Ehnfors M, Ekman I (2004). The Phenomena of participation and non-participation in Health Care-experiences of patients attending a nurse-led clinic for Chronic Heart Failure. Eur J Cardiovasc Nursing: J Working Group Cardiovasc Nurs Eur Soc Cardiol.

[54] Eldh AC, Ekman I, Ehnfors M (2006). Conditions for patient participation and non-participation in Health Care. Nurs Ethics.

[55] Tutton EMM (2005). Patient participation on a ward for frail older people. J Adv Nurs.

[56] Vahdat S, Hamzehgardeshi L, Hessam S, Hamzehgardeshi Z. Patient involvement in Health Care decision making: a review. Iran Red Crescent Med J 2014, 16(1). 10.5812/ircmj.12454 PMC3964421 24719703

[57] Koeck C. Imbalance of power between patients and doctors. Volume 349. British Medical Journal Publishing Group; 2014. 10.1136/bmj.g7485 25512331

[58] Baig AM, Humayaun A, Mehmood S, Akram MW, Raza SA, Shakoori T (2020). Qualitative exploration of factors associated with shared decision-making in Diabetes management: a healthcare provider’s perspective. Int J Qual Health Care.

[59] Matusitz J, Spear J (2015). Doctor-Patient Communication styles: a comparison between the United States and three Asian countries. J Hum Behav Social Environ.

[60] Obeidat R, Lally R (2018). Jordanian physicians’ perceived barriers and facilitators to patient participation in treatment decision-making: an exploratory study. Indian J Cancer.

[61] Mead EL, Doorenbos AZ, Javid SH, Haozous EA, Arviso Alvord L, Flum DR, Morris AM (2013). Shared decision-making for Cancer Care among racial and ethnic minorities: a systematic review. Am J Public Health.

[62] Bastiaens H, Van Royen P, Pavlic DR, Raposo V, Baker R (2007). Older people’s preferences for involvement in their own care: a qualitative study in primary healthcare in 11 European countries. Patient Educ Couns.

[63] Say R, Murtagh M, Thomson R (2006). Patients’ preference for involvement in medical decision making: a narrative review. Patient Educ Couns.

[64] Robinson JH, Callister LC, Berry JA, Dearing KA (2008). Patient-centered care and adherence: definitions and applications to improve outcomes. J Am Acad Nurse Pract.

[65] National Center for Health S. : Healthy People 2010 Final Review. In.; 2012.

[66] Howie JGR, Heaney D, Maxwell M (2004). Quality, core values and the general practice consultation: issues of definition, measurement and delivery. Fam Pract.

[67] Amin N, Cunningham SJ, Jones EM, Ryan FS (2020). Investigating perceptions of patient-centred care in orthodontics. J Orthodont.

[68] Peek ME, Wilson SC, Gorawara-Bhat R, Odoms-Young A, Quinn MT, Chin MH (2009). Barriers and facilitators to Shared decision-making among African-americans with Diabetes. J Gen Intern Medicine: JGIM.

[69] Aminaie N, Mirlashari J, Lehto R, Lashkari M, Negarandeh R (2019). Iranian Cancer patients perceptions of barriers to participation in Decision-Making: potential impact on patient-centered care. Asia-Pacific J Oncol Nurs.

[70] Abdulhadi N, Al Shafaee M, Freudenthal S, Ostenson C-G, Wahlström R (2007). Patient-provider interaction from the perspectives of type 2 Diabetes patients in Muscat, Oman: a qualitative study. BMC Health Serv Res.

[71] Brown RF, Butow PN, Henman M, Dunn SM, Boyle F, Tattersall MH (2002). Responding to the active and passive patient: flexibility is the key. Health Expect.

[72] Ward J, Cody J, Schaal M, Hojat M (2012). The empathy enigma: an empirical study of decline in empathy among undergraduate nursing students. J Prof Nurs.

[73] Pereira L, Figueiredo-Braga M, Carvalho IP (2016). Preoperative anxiety in ambulatory Surgery: the impact of an empathic patient-centered approach on psychological and clinical outcomes. Patient Educ Couns.

[74] Ghiyasvandian S, Abdolrahimi M, Zakerimoghadam M, Ebadi A (2018). Therapeutic communication of Iranian nursing students: a qualitative study. Pertanika J Social Sci Humanit.

[75] Moudatsou M, Stavropoulou A, Philalithis A, Koukouli S (2020). The role of Empathy in Health and Social Care professionals. Healthcare.

[76] Hasnain M, Connell KJ, Menon U, Tranmer PA (2011). Patient-centered care for Muslim women: Provider and patient perspectives. J Women’s Health.

[77] Tsianakas V, Liamputtong P (2002). What women from an Islamic background in Australia say about care in pregnancy and prenatal testing. Midwifery.

[78] Shibily FM, Aljohani NS, Aljefri YM, Almutairi AS, Almutairi WZ, Alhallafi MA, Alsharif F, Almutairi W, Badr H (2021). The perceptions of nurses and nursing students regarding family involvement in the care of hospitalized adult patients. Nursing reports. (Pavia Italy).

[79] Fernandes C, Gomes J, Martins MM, Gomes B, Gonçalves L (2015). The importance of families in nursing care: nurses’ attitudes in the hospital environment. Revista De Enfermagem referência.

[80] Hofstede G, Hofstede GJ, Minkov M. Cultures and organizations : software of the mind : intercultural cooperation and its importance for survival, Revised and expanded 3rd edition. New York: McGraw-Hill; 2010.

[81] Abouqal R, Phua J, Arabi YM (2018). Patient-physician relationship in specific cultural settings. Intensive Care Med.

